# Optimal Timing in Cervical Spinal Cord Injury: A Comprehensive Meta-Analysis of Ultra-Early Surgical Intervention Within Five Hours

**DOI:** 10.7759/cureus.62015

**Published:** 2024-06-09

**Authors:** Matthew T Carr, Pemla Jagtiani, Abhiraj D Bhimani, Mert Karabacak, Brian Kwon, Konstantinos Margetis

**Affiliations:** 1 Neurosurgery, Mount Sinai Hospital, New York, USA; 2 School of Medicine, State University of New York (SUNY) Downstate Health Sciences University, Queens, USA; 3 Neurosurgery, Icahn School of Medicine at Mount Sinai, New York, USA; 4 Neurosurgery, Mount Sinai Health System, New York, USA; 5 Neurosurgery, University of British Columbia, Vancouver, CAN

**Keywords:** cervical spine, meta-analysis, neurological recovery, surgical decompression, spinal cord injury

## Abstract

The optimal timing of surgery for cervical spinal cord injuries (SCI) and its impact on neurological recovery continue to be subjects of debate. This systematic review and meta-analysis aims to consolidate and assess the existing evidence regarding the efficacy of ultra-early decompression surgery in improving clinical outcomes after cervical SCI. A search was conducted in PubMed, Embase, Cochrane, and CINAHL databases from inception until September 18, 2023, focusing on human studies. The groups were categorized into ultra-early decompression (decompression surgery ≤ 5 hours post-injury) and a control group (decompression surgery between 5-24 hours post-injury). A random effects meta-analysis was performed on all studies using R Studio. Outcomes were reported as effect size (OR, treatment effect, and 95% CI. Of the 140 patients, 63 (45%) underwent decompression ≤ 5 hours, while 77 (55%) underwent decompression > 5 hours post-injury. Analysis using the OR model showed no statistically significant difference in the odds of neurological improvement between the ultra-early group and the early group (OR = 1.33, 95% CI: 0.22-8.18, p = 0.761). This study did not observe significant neurological improvement among cervical SCI patients who underwent decompression within five hours. Due to the scarcity of literature on the ultra-early decompression of cervical SCI, this study underscores the necessity for additional investigation into the potential benefits of earlier interventions for cervical SCI to enhance patient outcomes.

## Introduction and background

Traumatic spinal cord injury (SCI) is a catastrophic neurological event, with an annual incidence of 18,000 new SCI cases each year [1]. It has a profoundly devastating impact on personal, social, and economic levels, resulting in lifetime costs as high as 4.6 million dollars per patient [2]. Patients with complete cervical SCI suffer from various neurological impairments, including motor paralysis, loss of sensation, and potential respiratory impairment, which affect their ability to move and breathe [3].

Surgical decompression aims to improve neurological outcomes in patients with traumatic SCI. However, there is considerable debate about the timing of surgical intervention and its influence on neurological recovery. The Surgical Timing in Acute Spinal Cord Injury Study (STASCIS) trial provided support for early decompression, defined as within 24 hours of injury, improving neurological outcomes [4]. Another prospective study by Jug M et al. highlights superior neurological recovery with decompression performed within 8 hours of injury compared to decompression conducted 8 to 24 hours post-injury [5]. On the other hand, a retrospective cohort study by Mattiassich G et al. reported inferior neurological recovery with decompression performed within the first five hours compared to decompression within 5-24 hours [6]. Therefore, definitive guidelines have not yet been established regarding the optimal timing of surgical decompression for cervical SCI.

In this study, we conduct a systematic review and meta-analysis to evaluate neurological recovery in cervical SCI after surgical decompression performed within time frames shorter than 24 hours. While the timeframe constituting “ultra-early” decompression has not been defined, for the purposes of this review, we have defined it as within five hours.

## Review

Methods

Search Criteria

This study follows the Preferred Reporting Items for Systematic Reviews and Meta-Analyses (PRISMA) guidelines [7]. PubMed, Embase, Cochrane, and CINAHL databases were searched on September 18, 2023, and detailed search criteria can be found in Appendix 1.

Eligibility Criteria 

Inclusion criteria followed the Population, Intervention, Comparator, Outcomes, Study Design (PICOS) framework. The target population (P) was defined as patients of any age with cervical SCI. The intervention (I) involved surgical decompression, and comparators (C) were ultra-early decompression surgery, defined as surgery within 5 hours of injury, and early surgery performed between 5 to 24 hours. The outcome of interest (O) was an improvement in the American Spinal Injury Association Impairment Scale (AIS) or Frankel grade. The study design (S) included clinical trials and observational studies. Studies were excluded if they met the following exclusion criteria: (1) studies that did not include a separate group for those operated on within 5 hours, (2) full text not retrievable, (3) case reports, reviews, opinions, and (4) non-operative management.

Study Selection 

**Figure 1:**
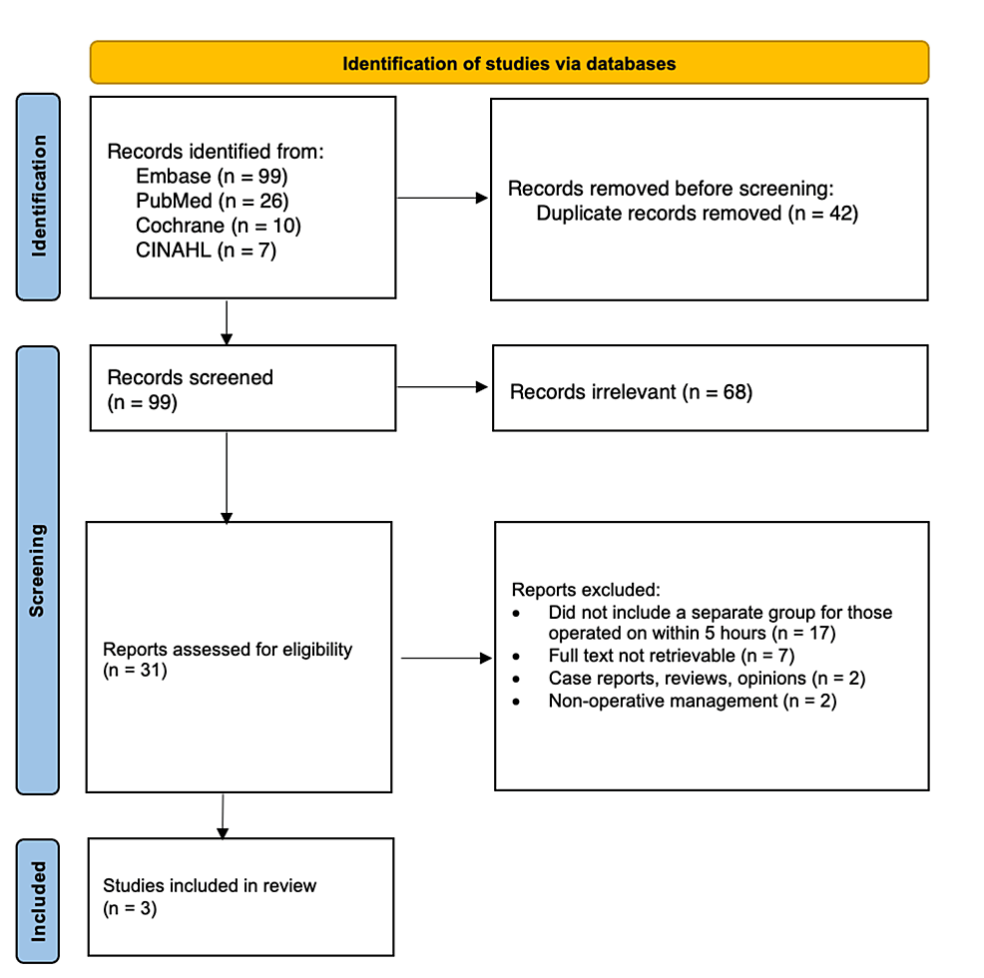
PRISMA flow chart. PRISMA: Preferred Reporting Items for Systematic Reviews and Meta-Analyses.

All identified citations were imported into the Covidence systematic review software [8]. Two independent reviewers (Author 1 and Author 2) screened titles and abstracts using the eligibility criteria. Subsequently, the independent reviewers (Author 1 and Author 2) analyzed the full texts of the articles to determine whether they met the inclusion criteria. Any disagreements were settled with the assistance of a third reviewer (Author 4). The PRISMA flow diagram in Figure 1 summarizes the selection process of the studies.

Data Collection 

Two reviewers (Author 1 and Author 2) independently extracted the data. The selected patients were divided into two groups: ultra-early surgery and early surgery. AIS/Frankel grades before and after surgery were collected. Improvement in the AIS/Frankel grade was assigned a corresponding numerical scale, where an improvement to a subsequent AIS/Frankel grade was given a numerical value of 1. Although the AIS/Frankel scale is ordinal, we treated it as a continuous variable. The Cochrane Handbook for Systematic Reviews of Interventions confirms that this is a viable option for the analysis of ordinal data [9]. While the AIS is more commonly used for grading neurological outcomes following SCI, the Frankel grade has also been used for the same purpose, as it similarly has a 5-grade classification system. We treated these scales as comparable ordinal scales for analysis.

Risk of Bias and Credibility of Evidence Assessments 

**Figure 2:**
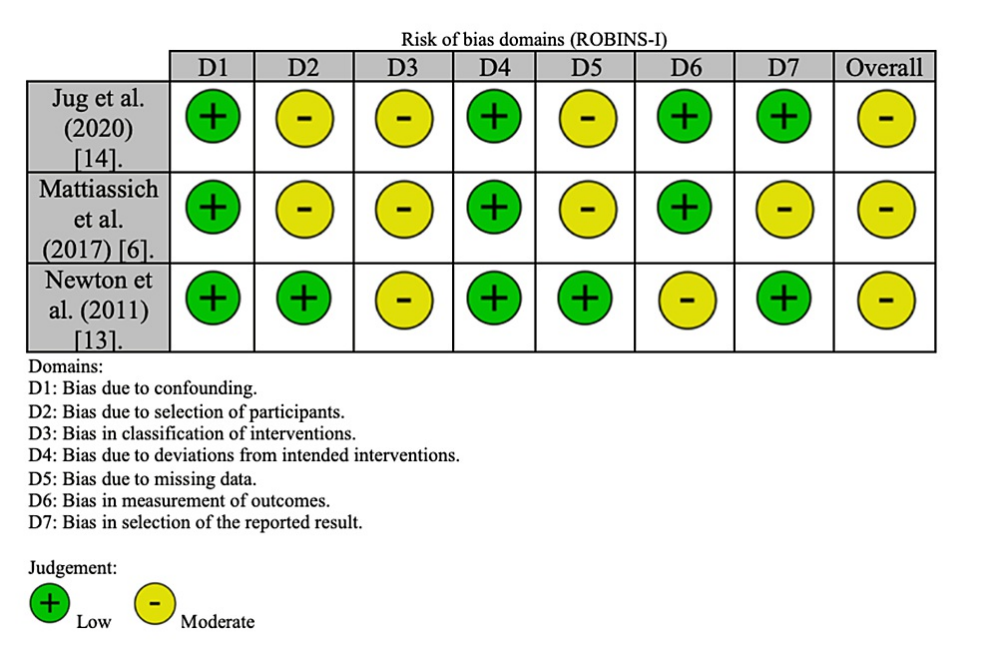
Graphical representation of risk of bias analysis analyzed by ROBINS-I. ROBIN-I: Risk of Bias in Non-randomised Studies - of Interventions.

The ROBINS-I tool was used to assess the risk of bias in the non-randomized studies (Figure 2). The tool uses "signaling questions" to address seven domains where bias could potentially be introduced [10]. The quality of evidence was evaluated based on the Grading of Recommendations Assessment, Development, and Evaluation (GRADE) system [11].

Meta-Analysis

All relevant extracted data were recorded in an Excel spreadsheet, and all analyses were conducted using R Studio Desktop version 2021.09.1 for Mac. The meta-analysis was performed using the metafor package in R to assess and compare ultra-early and early decompression. A random-effects model was chosen since it accounts for uncertainty due to heterogeneity among studies [12]. In comparing ultra-early versus early decompression, the effect size was reported as the OR and 95% CI. The analysis aimed to calculate a single effect size, proportion, and 95% CI. All effect sizes were displayed in the form of a forest plot. A p-value of less than 0.05 was considered statistically significant.

Results

One hundred forty patients were included from three studies (Table 1). Sixty-three (45%) underwent decompression ≤5 hours, while seventy-seven (55%) underwent decompression between 5 to 24 hours post-injury. One of the included studies, Newton D et al. [13], used a Frankel grade, rather than an AIS grade, but for the analysis, we treated these as similar ordinal scales for comparison.

**Table 1:** Summary of included studies. SCI: Spinal cord injuries; AIS: American Spinal Injury Association Impairment Scale.

Study	Type	Number of Patients	Key Points
Newton D et al. (2011) [13]	Retrospective case series	39	Patients with acute cervical SCI and facet dislocation when playing rugby. In ultra-early group 5 patients made a full recovery versus in early group 0 made full recovery. Permanent damage to SCI results from secondary injury but can be prevented if cervical facet dislocations are reduced within 4 hrs.
Mattiassich G et al. (2017) [6]	Retrospective cohort study	37	Patients presenting with cervical SCI at one of 6 Austrian trauma centres. A significantly higher AIS difference was observed in patients who underwent decompression > 5 hrs after trauma than those ≤ 5 hrs. Decompression of the spinal cord within 24 hrs after SCI was associated with an improved neurological outcome. No additional neurological benefit was observed in patients who underwent decompression ≤ 5 hrs.
Jug M et al. (2020) [14]	Prospective cohort study	64	Patients with acute cervical SCI and fracture or dislocation of the subaxial cervical spine from a level 1 trauma centre in Slovenia. The first 4-9 hrs represent a window of opportunity for surgical decompression with the best chances for neurologic recovery entailing ≥ 2 AIS grades 6 months after injury.

Jug M et al. performed a prospective cohort study on patients with acute cervical SCI and fractures or dislocations of the subaxial cervical spine [14]. This study states that the optimal timing of decompression is within the first 4-9 hours post-injury. They reported the number of patients with significant neurological recovery, defined as an improvement of at least two AIS grades, depending on how many hours after injury their decompression was performed. While this study only reported AIS ≥ 2, and < 2, it provided data for each hour, rather than simply reporting ≤ 5 hours and > 5 hours. The study included 18 patients who underwent surgery ≤ 5 hours and 46 patients > 5 hours.

Mattiassich G et al. performed a retrospective study of patients with isolated traumatic cervical SCI who underwent decompression over a 10-year period [6]. The study used categorical changes in AIS scores which were converted to numerical values. While this study provided the data to calculate the number of patients with AIS improvement ≥ 1, it did not provide more granular hourly data, but rather simply reported aggregate numbers of patients who underwent surgery ≤ 5 hours and > 5 hours. The study included 27 patients who underwent surgery ≤ 5 hours, and 10 patients > 5 hours.

Newton D et al. conducted a retrospective study on patients’ rugby injuries to the cervical spine over a 12-year period [13]. The study reported categorical changes in Frankel grade, which were converted to numerical values. It provided data to calculate the number of patients with Frankel improvement ≥ 1, or ≥ 2 and included more granular hourly data as well. The Frankel grade also has a 5-grade classification system like that of AIS used to assess neurological improvement, but AIS is a newer version of Frankel grade that utilizes 'sacral sparing' to determine neurologically complete vs incomplete injury [15]. The study included 18 patients who underwent decompression ≤ 5 hours, and 21 patients > 5 hours.

Risk of Bias Assessment 

A risk of bias assessment was conducted for all the studies using the ROBINS-I tool, as they were all non-randomized studies. The assessment concluded that they had an overall moderate risk of bias (Figure 2). An analysis of the evidence profile using the GRADE system is presented in Table 2. The GRADE assessment offers a clear framework for presenting evidence summaries and employs a systematic approach to formulate clinical practice recommendations.

**Table 2:** GRADE evidence profile for this systematic review and meta-analysis GRADE: Grading of Recommendations Assessment, Development and Evaluation.

Study Limitations	Directness	Consistency	Precision	Reporting bias
High	Direct	Consistent	Precise	Undetected

Neurological Improvement 

**Figure 3:**
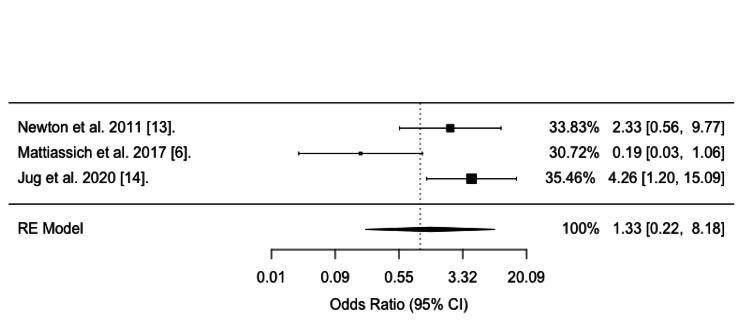
Forest plot of all three studies comparing the ultra-early group (≤ 5 hrs) with the early group (5-24 hrs), defining neurological improvement as an increase of ≥ 2 grades.

In a random-effects meta-analysis encompassing three studies, the odds of neurological improvement did not statistically differ between the ultra-early group (decompression ≤5 hours) and the early group (decompression 5-24 hours) ((OR = 1.33, 95% CI: 0.22-8.18), p = 0.7609), as illustrated in Figure 3. The analysis defined AIS/Frankel improvement as ≥ 2, as it incorporated the Jug M et al. study [14], which exclusively reported data using this criterion.

Discussion 

Determining Ultra-Early Timing 

Current guidelines underscore the importance of early surgical decompression (<24 hours) following SCI as it is associated with improved sensorimotor recovery [16]. Du et al. have provided specific recommendations based on the type of AOSpine subaxial cervical SCI. Their results indicated that type A and F1-3 fractures do not require aggressive early decompression. However, early decompression for type B and type C/F4 fractures may lead to improved clinical outcomes [17]. The primary aim of urgent decompression in SCI is to reinstate blood flow and spinal cord perfusion pressure, thereby effectively halting the progression of secondary injury. Although the 24-hour time frame was arbitrarily set, there is a logical basis for investigating whether surgical decompression at an even earlier stage could offer additional benefits [18]. In other neurological emergencies such as subdural hematoma or ischemic infarct, far earlier time points have been established for surgical intervention leading to improved outcomes [19,20]. Both subdural hematoma and ischemic infarct exert pressure on the brain, while cervical SCI results in compression of the spinal cord. As a result, the level of surgical urgency and the earlier time points relevant to other neurological emergencies can also be considered applicable to SCI.

There is inconsistency in the relevant literature regarding the definition of ultra-early decompression, with the time threshold spanning from 4 to 12 hours. There is also inconsistency regarding the true improvement of outcomes following ultra-early decompression. Biglari B et al. categorize all SCI collectively, characterizing ultra-early decompression as occurring within a 4-hour timeframe. Their findings do not indicate any enhanced neurological function compared to treatments between 4 and 24 hours after injury [21]. Aarabi B et al. define ultra-early cervical SCI as within 12 hours and also do not report improved outcomes compared to treatment within 12-24 hours [18]. Multiple recent meta-analyses have found improved outcomes with decompression within eight hours [22,23]. Drawing on the clinical and academic experience of the authors, a 5-hour time window could be a clinically feasible target for ultra-early decompression in certain institutions, depending on their pattern of emergency referrals.

Neurological Improvement 

The International Standards for Neurological Classification of SCI (ISNCSCI) is an internationally accepted standard for classifying neurological impairment. It provides a standardized method of completing the neurological assessment and then classifying the SCI and completeness of injury. Before the adoption of the ISNCSCI, the Frankel scale was predominantly utilized for categorizing SCI [24].

This meta-analysis comprised three studies, totaling 140 patients, that reported neurological outcomes after ultra-early (≤5 hours) decompressive surgery for cervical SCI. When all three studies were pooled, no statistically significant difference in the odds of neurological improvement between the ultra-early and early groups was found.

It is important to acknowledge that even if compelling evidence in favor of ultra-early decompression for cervical SCI were presented, there are formidable challenges associated with its implementation. A study of patients in the North American Clinical Trials Network from 2005 to 2019 revealed that only 40.5% of patients with cervical SCI were operated on within 12 hours of injury [25]. Reasons for the delay included medical stabilization, transportation from the scene to a hospital with appropriate trauma services, radiographic and laboratory evaluation, surgical decision-making, and operating room availability [25]. In less developed regions, financial constraints, equipment accessibility issues, and the absence of multidisciplinary teams further complicate matters. It is worth noting that of the three included studies, two are from Europe and one from South Africa. The Newton study also dealt with dislocations managed by closed reduction, which requires substantially less time to plan and perform than surgical laminectomy and fusion, thus avoiding some of the barriers to ultra-early surgical decompression [13]. Nevertheless, if a substantial body of convincing research emerges supporting the effectiveness of ultra-early decompression in enhancing neurological recovery, our practices must adapt to meet the demand while combating the anticipated challenges.

Limitations

It is crucial not to disregard several limitations in this meta-analysis. We are aware that a meta-analysis involving only three studies may lack statistical power. Moreover, of these three, only the paper by Jug M et al. evaluated the association of decompression timing and adjusted for the degree of spinal cord compromise and severity of cervical SCI, leaving this as a significant confounding variable [14]. Further studies are required to better understand the interrelationships among injury severity, timing of decompression, and its effect on neurological outcomes in cervical SCI. For example, it may be that incomplete injuries resulting from lower energy traumas are the most likely to benefit from ultra-early surgery. Defining such sub-populations would be an important task for future research. Another limitation lies in the limited available research, where inconsistency arises regarding the definition of ultra-early, with timing spanning from 4 to 12 hours. There is also inconsistency in how the data is reported within the included articles. Jug M et al. limit AIS improvement to ≥ 2, so any analysis that includes this study must also only define AIS improvement as ≥ 2 for consistency [14]. This may have caused our analysis to miss any significant difference in 1-grade improvement. The Newton D et al. study reports neurological improvement using the Frankel scale, while the other two studies use the AIS grade [13]. This inconsistency not only introduces ambiguity but also imposes significant limitations. The Frankel classification scale lacks specificity regarding the level of spine injury, and it fails to articulate distinctions between 'motor useful' and 'motor useless' grades, resulting in subjective grading [24]. While there are obviously noteworthy differences between the AIS and Frankel grading systems, they are comparable in their gradation from complete injury (A) to normal function (E), and we felt the inclusion of Frankel grade would be important to avoid missing studies that predate the AIS grade. One crucial aspect to bear in mind is that for a patient to undergo “ultra-early” surgery, the individual must have also had a baseline neurological examination conducted at an “ultra-early” time point. It is recognized that such an ultra-early examination may not reliably represent the true severity of the injury [26]. Patients who are AIS C or D incomplete SCI at 24 hours post-injury may appear as an AIS A complete SCI if they are examined immediately at the scene of the accident or upon arrival at the hospital. Consequently, deciphering whether such individuals experience improvement as a result of ultra-early surgery or if they were destined for significant spontaneous recovery becomes quite challenging. This issue indeed complicates the interpretation of the effect of any study on the timing of ultra-early surgical decompression, given that patients who have earlier surgery are inherently more likely to have earlier examinations.

## Conclusions

The limited research on ultra-early decompression for cervical SCI underscores the importance of consolidating and analyzing data from available studies. This meta-analysis reveals no significant neurological improvement among cervical SCI patients who undergo decompression within 5 hours. However, the current research and data on five-hour decompression for cervical SCI remain quite limited, highlighting the need for future research in this area.

## Appendices

Appendix 1

PubMed: 

("Immediate"[Title/Abstract] OR "early"[Title/Abstract] OR "ultra-early"[Title/Abstract] OR "5 hours"[Title/Abstract] OR "five hours"[Title/Abstract] OR "Immediate"[Title/Abstract] OR "prompt"[Title/Abstract]) AND ("surgical management"[Title/Abstract] OR "surgical decompression"[Title/Abstract] OR "stabilisation"[Title/Abstract] OR "stabilization"[Title/Abstract] OR "operative management"[Title/Abstract] OR "decompressive surgery"[Title/Abstract] OR "operative timing"[Title/Abstract]) AND ("spinal cord injury"[Title/Abstract] AND "acute"[Title/Abstract] AND "cervical"[Title/Abstract] AND "traumatic"[Title/Abstract]) AND ("neurological outcomes"[Title/Abstract] OR "recovery"[Title/Abstract] OR "ASIA"[Title/Abstract] OR "improvement"[Title/Abstract])

EMBASE, COCHRANE, CINAHL databases:

("Immediate" OR "early" OR "ultra-early" OR "5 hours" OR "five hours" OR "Immediate" OR "prompt") AND ("surgical management" OR "surgical decompression" OR "stabilisation" OR "stabilization" OR "operative management" OR "decompressive surgery" OR "operative timing") AND ("spinal cord injury" AND "acute" AND "cervical" AND "traumatic") AND ("neurological outcomes" OR "recovery" OR "ASIA" OR "improvement").

## References

[1] Jain NB, Ayers GD, Peterson EN, Harris MB, Morse L, O'Connor KC, Garshick E (2015). Traumatic spinal cord injury in the United States, 1993-2012. JAMA.

[2] Ahuja CS, Martin AR, Fehlings M (2016). Recent advances in managing a spinal cord injury secondary to trauma. F1000Res.

[3] Shakil H, Malhotra AK, Jaffe RH (2023). Factors influencing withdrawal of life-supporting treatment in cervical spinal cord injury: a large multicenter observational cohort study. Crit Care.

[4] Fehlings MG, Vaccaro A, Wilson JR (2012). Early versus delayed decompression for traumatic cervical spinal cord injury: results of the Surgical Timing in Acute Spinal Cord Injury Study (STASCIS). PLoS One.

[5] Jug M, Kejžar N, Vesel M, Al Mawed S, Dobravec M, Herman S, Bajrović FF (2015). Neurological recovery after traumatic cervical spinal cord injury is superior if surgical decompression and instrumented fusion are performed within 8 hours versus 8 to 24 hours after injury: a single center experience. J Neurotrauma.

[6] Mattiassich G, Gollwitzer M, Gaderer F (2017). Functional outcomes in individuals undergoing very early(<5 h) and early (5-24 h) surgical decompression in traumatic cervical spinal cord injury: analysis of neurological improvement from the Austrian Spinal Cord Injury Study. J Neurotrauma.

[7] Page MJ, McKenzie JE, Bossuyt PM (2021). The PRISMA 2020 statement: an updated guideline for reporting systematic reviews. BMJ.

[8] Veritas Health Innovation (2023). Veritas Health Innovation. https://www.covidence.org.

[9] Higgens J, Thomas J, Chandler J, Cumpston M, Li T, Page M, Welch V (2019). Cochrane Handbook for Systematic Reviews of Interventions.

[10] Sterne JA, Hernán MA, Reeves BC (2016). ROBINS-I: a tool for assessing risk of bias in non-randomised studies of interventions. BMJ.

[11] Guyatt GH, Oxman AD, Vist GE, Kunz R, Falck-Ytter Y, Alonso-Coello P, Schünemann HJ (2008). GRADE: an emerging consensus on rating quality of evidence and strength of recommendations. BMJ.

[12] Dettori JR, Norvell DC, Chapman JR (2022). Fixed-effect vs random-effects models for meta-analysis: 3 points to consider. Global Spine J.

[13] Newton D, England M, Doll H, Gardner BP (2011). The case for early treatment of dislocations of the cervical spine with cord involvement sustained playing rugby. J Bone Joint Surg Br.

[14] Jug M, Kejžar N, Cimerman M, Bajrović FF (2019). Window of opportunity for surgical decompression in patients with acute traumatic cervical spinal cord injury. J Neurosurg Spine.

[15] Kirshblum S, Botticello A, Benedetto J, Donovan J, Marino R, Hsieh S, Wagaman N (2020). A comparison of diagnostic stability of the ASIA impairment scale versus Frankel classification systems for traumatic spinal cord injury. Arch Phys Med Rehabil.

[16] Badhiwala JH, Wilson JR, Witiw CD (2021). The influence of timing of surgical decompression for acute spinal cord injury: a pooled analysis of individual patient data. Lancet Neurol.

[17] Du JP, Fan Y, Zhang JN, Liu JJ, Meng YB, Hao DJ (2019). Early versus delayed decompression for traumatic cervical spinal cord injury: application of the AOSpine subaxial cervical spinal injury classification system to guide surgical timing. Eur Spine J.

[18] Aarabi B, Akhtar-Danesh N, Chryssikos T (2020). Efficacy of ultra-early (<12 h), early (12-24 h), and late (>24-138.5 h) surgery with magnetic resonance imaging-confirmed decompression in American Spinal Injury Association Impairment Scale grades A, B, and C cervical spinal cord injury. J Neurotrauma.

[19] Díez-Tejedor E, Fuentes B (2004). Acute care in stroke: the importance of early intervention to achieve better brain protection. Cerebrovasc Dis.

[20] Oh CH, Shim YS, Yoon SH, Hyun D, Park H, Kim E (2016). Early decompression of acute subdural hematoma for postoperative neurological improvement: a single center retrospective review of 10 years. Korean J Neurotrauma.

[21] Biglari B, Child C, Yildirim TM, Swing T, Reitzel T, Moghaddam A (2016). Does surgical treatment within 4 hours after trauma have an influence on neurological remission in patients with acute spinal cord injury?. Ther Clin Risk Manag.

[22] Bhimani AD, Carr MT, Al-Sharshai Z, Hickman Z, Margetis K (2023). Ultra-early (≤8 hours) surgery for thoracolumbar spinal cord injuries: a systematic review and meta-analysis. N Am Spine Soc J.

[23] Ma Y, Zhu Y, Zhang B, Wu Y, Liu X, Zhu Q (2020). The impact of urgent (<8 hours) decompression on neurologic recovery in traumatic spinal cord injury: a meta-analysis. World Neurosurg.

[24] Roberts TT, Leonard GR, Cepela DJ (2017). Classifications in brief: American Spinal Injury Association (ASIA) Impairment Scale. Clin Orthop Relat Res.

[25] Neal CJ, Ugiliweneza B, Toups EG (2023). Variability in early surgery for acute cervical spinal cord injury patients: an opportunity for enhanced care delivery. J Neurotrauma.

[26] Evaniew N, Sharifi B, Waheed Z (2020). The influence of neurological examination timing within hours after acute traumatic spinal cord injuries: an observational study. Spinal Cord.

